# Clinical and Etiological Profile of Children With Acute Viral Encephalitis in a Tertiary Care Hospital: A Cross-Sectional Study

**DOI:** 10.7759/cureus.66588

**Published:** 2024-08-10

**Authors:** Amulya Dharmagadda, Sampada Tambolkar, Shailaja V Mane, Sneha Singh

**Affiliations:** 1 Pediatrics, Dr. D. Y. Patil Vidyapeeth, Pune, Pune, IND

**Keywords:** pediatric intensive care unit (picu), morbidity and mortality, public health issue, epidemiological trends, viral etiology, acute encephalitis syndrome (aes)

## Abstract

Background: Acute encephalitis refers to the clinical diagnosis of children who have a sudden onset of symptoms and show evidence of inflammatory lesions in the brain. Timely diagnosis is crucial for both lifesaving measures and the preservation of brain functions.

Objective: The objective of the study was to determine the clinical and etiological profile of acute viral encephalitis in children within a tertiary care hospital.

Methods: This hospital-based cross-sectional study was conducted in the Pediatric Intensive Care Unit (PICU) at Dr. D. Y. Patil Medical College, Hospital, and Research Centre in Pune. The study included children aged one month to 12 years diagnosed with suspected viral encephalitis. Over 22 months, from August 2022 to June 2024, 35 children who met the inclusion criteria were enrolled. Data collection involved clinical examinations, laboratory investigations, and imaging studies, following informed consent from the parents or guardians.

Results: The study examined 35 patients with suspected acute encephalitis syndrome (AES) and found a male-to-female ratio of 3.4:1. Among the patients, 22 (62.85%) had a confirmed viral etiology, while 13 (37.17%) had an unknown etiology. The most common virus isolated was mumps, with school-age children most affected. The cases were concentrated in the Chikhali, Bhosari, Nigdi, and Chinchwad regions. Symptoms included fever, seizures, vomiting, and altered mental status. Low vaccination rates were observed, and the Glasgow Coma Scale (GCS) scores, shock incidence, and ventilation showed an association with mortality. Most patients required intensive care, antiedema measures, antibiotics, and antivirals. The mortality rate was 11.4%, with 17% of patients discharged with neurological sequelae.

Conclusion: Causative agents such as mumps, herpes simplex virus (HSV), dengue, and many other viruses are now more prevalent than the Japanese encephalitis (JE) virus. Bad clinical course and fatal outcomes are observed in patients affected with rabies, HSV, and H1N1 influenza virus. Factors such as GCS scores, shock, and need for ventilation play a significant role in determining patient prognosis. Early detection and prompt treatment may aid in better outcomes for patients.

## Introduction

Acute encephalitis syndrome (AES) is a major public health issue in India, known for its high rates of morbidity and mortality. According to the World Health Organization (WHO), acute encephalitis syndrome is characterized by a sudden onset of fever, changes in mental status, such as confusion or coma, and/or new seizures [1].

Acute encephalitis syndrome prevalence varies, averaging from 3.5 to 7.4 cases per 100,000 people each year, and it is more common in children [2]. Although viruses are often responsible, identifying the specific cause is challenging due to diagnostic difficulties and similar central nervous system (CNS) illnesses. From 2008 to 2014, India saw over 44,000 acute encephalitis syndrome cases and nearly 6,000 deaths, particularly in Uttar Pradesh and Bihar [3]. In 2016, an outbreak in Gorakhpur resulted in over 125 child deaths in one hospital [4].

The Japanese encephalitis (JE) virus is a major cause of AES in India, with 1,500-4,000 cases annually [5]. Other viruses, including enteroviruses, Epstein-Barr virus (EBV), and herpes simplex virus (HSV), also contribute. The Chandipura virus, spread by mosquitoes and sandflies, is prevalent in India. Despite extensive research, the cause of acute-onset fever and altered sensorium often remains unclear in states such as Uttar Pradesh, Bihar, and West Bengal [6].

Addressing acute encephalitis syndrome requires better surveillance, diagnostics, vaccination, and vector control. Research and improved healthcare infrastructure are vital to reducing its impact. Our study aims to determine the clinical and etiological profile of acute viral encephalitis in children in a tertiary care hospital.

## Materials and methods

Study design and data collection

This was a cross-sectional study conducted in the Paediatric Intensive Care Unit (PICU) at Dr. D. Y. Patil Medical College, Hospital, and Research Centre in Pimpri, Pune. The study was approved by the Institutional Ethics Subcommittee of Dr. D. Y. Patil Vidyapeeth, Pune (IESC/PGS/2022/25). A total of 35 children aged one month to 12 years who met the inclusion criteria were included. It was conducted over 22 months, from August 1, 2022, to June 30, 2024. Before sample collection, patient/parental consent was obtained for routine investigations and also for special investigations such as lumbar puncture and radio imaging. Data collection included demographic information, clinical data at admission, and in-hospital clinical data and investigations, outcomes at the time of discharge, and short-term follow-ups at two weeks, one month, and three months.

Inclusion and exclusion criteria

Inclusion criteria included children presenting with symptoms of AES (fever, convulsions, and altered sensorium) and cerebrospinal fluid (CSF) analysis indicating viral encephalitis and parents who provided written informed consent. Exclusion criteria included children presenting with AES symptoms but with a clinical-investigational diagnosis confirmative of bacterial, tubercular, or fungal meningitis, non-infectious encephalopathy, or similar conditions (cerebral abscess, cerebral hemorrhage, infarction, Reye's syndrome, and metabolic or toxic encephalopathy), and children whose parents/caretakers did not provide informed consent.

Sample size calculation

Considering the feasibility of the study, according to the PICU registers and previous studies conducted, the suspected cases of viral encephalitis patient load was found to be 33 over a period of two years. Hence, the sample size was selected as 35.

Laboratory testing

Routine investigations such as a complete blood count, peripheral blood smear, red blood cell (RBC) indices, and blood sugar were performed on all subjects. Lumbar puncture was performed under aseptic precautions. CSF was collected and analyzed for cytology, biochemical markers, gram staining, acid-fast bacillus (AFB) staining, and culture/sensitivity. Viral culture and multiplex polymerase chain reaction (PCR) were used for virus isolation. Acute-phase samples were transported to the National Institute of Virology (NIV), Pune, in an ice box as soon as possible. Special investigations were performed as needed based on the case history, urine, throat swab, stool PCR, nasopharyngeal swab, and neuroimaging.

Statistical analysis

Data were recorded in a pre-designed study proforma, and data entry was done in a Microsoft Excel spreadsheet (Microsoft Corp., Redmond, WA). Statistical analysis was done using SPSS version 25 (IBM SPSS Statistics, Armonk, NY). Categorical variables were represented in the form of frequencies and percentages, and cross-tabulations were done to compare column proportions. Comparison was done using the Chi-square test. Distribution was represented by pie charts or bar graphs. Continuous variables were expressed in descriptive statistics tables as means, standard deviation, minimum and maximum values, and means, which were compared using an independent sample t-test. P<0.05 was considered significant, and P<0.01 was considered highly significant.

## Results

A total of 35 patients who were included in the study were assessed clinically and, on the basis of a CSF examination, were considered to have suspected AES of viral etiology and further studied.

**Figure 1:**
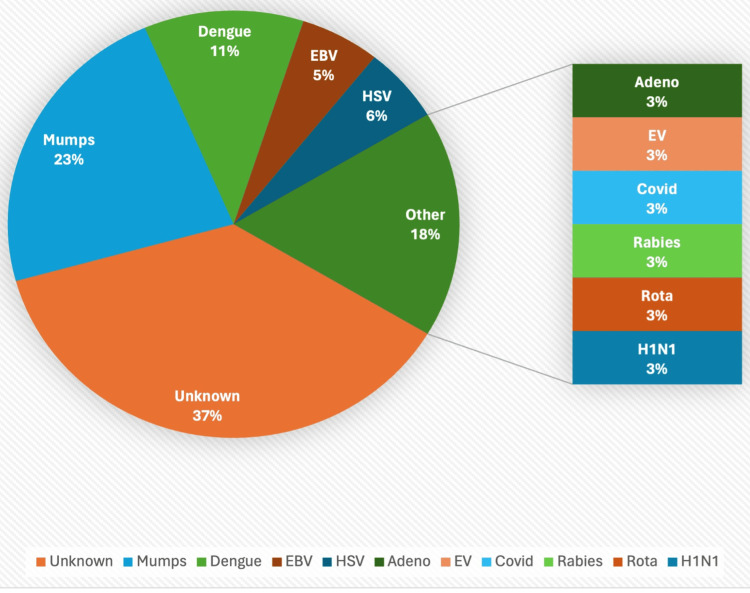
Percentage of different viral isolates in the study group EV: enterovirus, COVID: coronavirus disease, EBV: Epstein-Barr virus, HSV: herpes simplex virus

Out of these 35 patients, 22 (62.85%) patients had viral etiology, and 13 (37.17%) patients had unknown etiology, showing a male preponderance with 27 (77.1%) males compared to eight (22.9%) females, resulting in a gender ratio of approximately 3.4 males for every female. The percentage of viruses isolated in the study group is shown in Figure 1.

**Figure 2:**
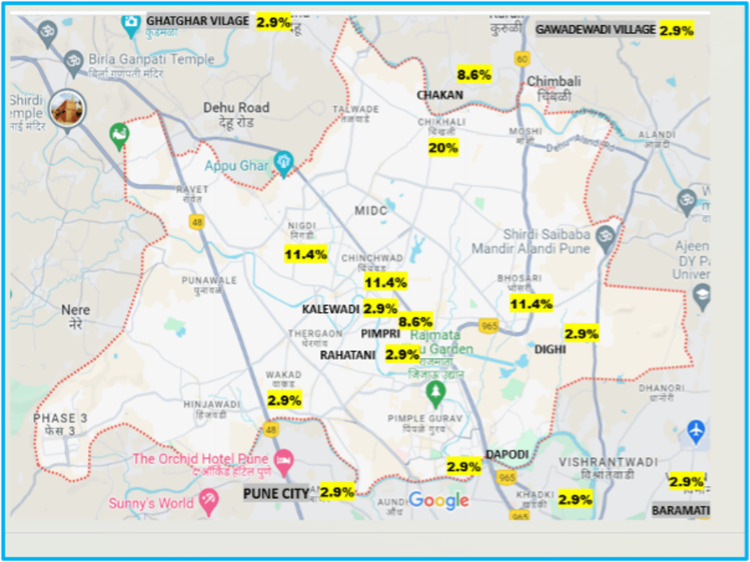
Geographical distribution of viral encephalitis cases in the PCMC area The area map was extracted from Google Maps and designed by the authors. PCMC: Pimpri Chinchwad Municipal Corporation

School-age children (>4 years) were the most affected, followed by infants and toddlers. The majority of cases were from the Chikhali region, followed by Bhosari, Nigdi, and Chinchwad, as shown in Figure 2.

The common clinical features of the study subjects are described in Table 1. Fever along with seizures, vomiting, and altered mental status are the most common presentations found. Other symptoms such as cheek swelling, rashes, and loose stools were found to be seen with only certain viral etiologies (Table 1).

**Table 1:** Distribution of participants based on symptoms according to various etiologies of viral encephalitis *Statistically significant The Chi-square test of independence is used to calculate the P value. EV: enterovirus, COVID: coronavirus disease, EBV: Epstein-Barr virus, HSV: herpes simplex virus

Clinical presentation	Etiology											Total (N=35)	Chi-square value and P value
	Covid (n=1)	Dengue (n=4)	Enterovirus (n=1)	H1N1 (n=1)	HSV (n=2)	Mumps (n=8)	Rabies (n=1)	Rotavirus (n=1)	EBV (n=2)	Adenovirus (n=1)	Unknown (n=13)		
Seizures	1 (100%)	2 (50%)	1 (100%)	1 (100%)	2 (100%)	4 (50%)	1 (100%)	1 (100%)	2 (100%)	0 (0%)	7 (53.8%)	22 (62.9%)	9.056 and 0.6
High-grade fever	1 (100%)	4 (100%)	1 (100%)	1 (100%)	1 (100%)	7 (88%)	1 (100%)	0 (0%)	2 (100%)	1 (100%)	13 (100%)	32 (91.4%)	2.471 and 0.07
Vomiting	0 (0%)	4 (100%)	1 (100%)	1 (100%)	0 (0%)	3 (38%)	0 (0%)	1 (100%)	0 (0%)	1 (100%)	8 (61.5%)	19 (54.3%)	3.286 and 0.13
Severe headache	0 (0%)	1 (25%)	0 (0%)	0 (0%)	0 (0%)	7 (88%)	0 (0%)	0 (0%)	0 (0%)	1 (100%)	6 (46.2%)	15 (42.9%)	18.833 and 0.13
Altered mental status	0 (0%)	4 (100%)	0 (0%)	1 (100%)	1 (50%)	2 (25%)	1 (100%)	0 (0%)	1 (50%)	1 (100%)	7 (54%)	18 (51.4%)	9.488 and 0.28
Cheek swelling	0 (0%)	0 (0%)	0 (0%)	0 (0%)	0 (0%)	8 (100%)	0 (0%)	0 (0%)	0 (0%)	0 (0%)	0 (0%)	8 (22.9%)	1.564 and <0.01*
Skin rashes	0 (0%)	2 (50%)	0 (0%)	0 (0%)	0 (0%)	0 (0%)	0 (0%)	0 (0%)	0 (0%)	0 (0%)	0 (0%)	2 (5.7%)	8.00 and 0.05*
Loose stools	0 (0%)	0 (0%)	1 (100%)	0 (0%)	0 (0%)	0 (0%)	1 (100%)	1 (100%)	0 (0%)	0 (0%)	0 (0%)	3 (8.6%)	17.429 and 0.002*
Refusal to feed	0 (0%)	0 (0%)	1 (100%)	0 (0%)	0 (0%)	0 (0%)	1 (100%)	1 (100%)	1 (50%)	0 (0%)	3 (23.1%)	7 (20%)	8.00 and 0.03*
Respiratory symptoms	1 (100%)	1 (25%)	0 (0%)	1 (100%)	0 (0%)	0 (0%)	0 (0%)	0 (0%)	0 (0%)	0 (0%)	0 (0%)	3 (8.6%)	6.057 and 0.09
Abdominal pain	0 (0%)	0 (0%)	0 (0%)	0 (0%)	0 (0%)	1 (12.5%)	0 (0%)	0 (0%)	0 (0%)	0 (0%)	2 (15.4%)	3 (8.6%)	5.343 and 0.19
Slurred speech	0 (0%)	0 (0%)	0 (0%)	0 (0%)	0 (0%)	1 (12.5%)	0 (0%)	0 (0%)	0 (0%)	0 (0%)	1 (7.7%)	2 (5.7%)	0.267 and 0.72
Ear pain	0 (0%)	0 (0%)	0 (0%)	0 (0%)	0 (0%)	0 (0%)	0 (0%)	0 (0%)	0 (0%)	0 (0%)	1 (7.7%)	1 (2.9%)	0.667 and 0.43

Vaccination rates were low, with only 26 (74.2%) patients receiving polio, rotavirus, and measles and rubella (MR) vaccines, and none of the patients received the measles, mumps, and rubella (MMR) vaccine or the JE vaccine. There was a significant statistical correlation between the Glasgow Coma Scale (GCS) score and the mortality of patients. Also, the need for mechanical ventilation and the incidence of shock at the time of admission showed a significant impact on outcomes (Table 2).

**Table 2:** Correlation between GCS score, shock, need for ventilation, and outcome of the viral encephalitis patients Mild to moderate: 9-15, severe: ≤8 The Chi-square test is used to see statistical significance. GCS: Glasgow Coma Scale

Parameter		Outcome		Total (N=35)	Chi-square value	P value
		Died (n=4)	Discharged (n=31)			
GCS score	Mild to moderate	1 (25%)	25 (80.6%)	26 (74.3%)	5.7429	0.017
	Severe	3 (75%)	6 (19.4%)	9 (25.7%)		
Shock	Yes	3 (75%)	9 (29%)	12 (34.3%)	3.3327	0.068
	No	1 (25%)	22 (71%)	23 (65.7%)		
Need for ventilation	Yes	4 (100%)	7 (22.6%)	11 (31.4%)	9.853	0.002
	No	0 (0%)	24 (77.4%)	24 (68.6%)		

Meningeal signs were the most common CNS findings. Cranial nerve palsies were observed in five patients. Papilledema was noted in two patients, and focal neurological deficits were identified in six patients, with two patients having hemiparesis, one having involuntary movements, and three having speech abnormalities.

Table 3 illustrates the CSF analysis of the patients. Lymphocytic predominance was observed in all patients.

**Table 3:** Distribution of CSF parameters of participants with acute viral encephalitis CSF: cerebrospinal fluid, TLC: total leukocyte count, SD: standard deviation

Statistics	Glucose (mg/dL)	Protein (g/L)	TLC
Mean	63.67	84.18	94.75
SD	16.65	92.05	239.668763
Minimum	28	16.70	6
Maximum	100	536	990

All patients tested negative for the CSF cartridge-based nucleic acid amplification test (CBNAAT), and no growth was detected in the CSF culture. Various methods used in the study to isolate viruses are mentioned in Table 4.

**Table 4:** Diagnostic methods used to detect various viral etiologies of participants with AES Numbers in parentheses: number of cases detected PCR: polymerase chain reaction, RT-PCR: real-time reverse transcriptase-polymerase chain reaction, VCA: viral capsid antigen, CSF: cerebrospinal fluid, HSV: herpes simplex virus, EBV: Epstein-Barr virus, COVID-19: coronavirus disease 2019

Virus isolated	Number of patients (N=35)	Diagnostic criteria
Mumps	8 (22.8%)	CSF PCR positive (8), throat swab PCR positive (5), serum PCR positive (3)
Dengue	4 (11.4%)	Positive dengue serology IgM (4)
HSV	2 (5.7%)	CSF PCR positive (2)
EBV	2 (5.7%)	Positive serology - EBV IgM antibody to VCA is positive (2)
Adenovirus	1 (2.8%)	CSF PCR positive (1)
H1N1	1 (2.8%)	Throat swab PCR - influenza A positive (1)
Rotavirus	1 (2.8%)	Stool PCR positive (1)
COVID-19	1 (2.8%)	Serology positive for COVID-19 IgM and RT-PCR was positive (1)
Enterovirus	1 (2.8%)	Stool PCR positive (1)
Rabies	1 (2.8%)	CSF antibodies detected (1)

Fourteen (40%) subjects showed hyperintensified lesions in the subcortical region of the magnetic resonance imaging (MRI) of the brain, while seven (20%) patients showed cortical involvement, five (14.2%) patients showed both cortical and subcortical involvement, and six (17.1%) patients showed normal results (Table 5).

**Table 5:** MRI findings of all participants included in this study MRI: magnetic resonance imaging, HSV: herpes simplex virus, COVID: coronavirus disease, EBV: Epstein-Barr virus

MRI findings	Etiology	Frequency
Mumps encephalitis	Normal	5
	Not done given the metallic implant	2
	Frontal parietal subcortical white matter affected	1
Unknown etiology	Subcortical region: basal ganglia, thalamus, and hippocampus	5
	Both cortical and subcortical involvement	2
	Cortical involvement: frontoparietal region	3
	Cortical involvement: frontotemporal region	2
	Cortical involvement: frontoparietal and parietal occipital lobe	1
Dengue encephalitis	Hyperintensity area of restricted diffusion, cerebral edema present	1
	Cortex and subcortical white matter in bilateral cerebral and cerebellar hemispheres and leptomeningeal enhancement along bilateral supratentorial brain parenchyma	1
	Swollen and edematous bilateral thalami	1
	Microhemorrhages in thalami, subcortical deep matter periventricular matter	1
Enterovirus encephalitis	Thalamic involvement	1
H1N1 encephalitis	Heterogenous patchy post-contrast enhancement and hemorrhagic changes	1
HSV encephalitis	Deep and subcortical white matter of bilateral cerebral and cerebellar hemispheres, peduncles, and internal capsules affected	1
	Medial temporal lobes, inferolateral frontal lobes affected	1
Rabies encephalitis	Bilateral peri-trigonal white matter and bilateral deep frontal white matter, non-specific white matter changes, cerebellum and ventral aspect of the pons affected with cerebral atrophy	1
Rotavirus encephalitis	Bilateral periventricular white matter and centrum semi-ovale	1
COVID encephalitis	Lentiform nucleus and hippocampus affected	1
Adenovirus encephalitis	Normal	1
EBV encephalitis	Predominantly involving splenium, bilateral periventricular white matter, bilateral parietal white matter, and bilateral corona radiata	1
	Right medial temporal lobe and left caudate nucleus	1

The electroencephalogram (EEG) showed epileptiform discharges in four patients and a slowing of waves in two patients.

Thirty-five (100%) patients needed PICU admission, antiedema, antibiotics, and acyclovir, followed by steroids and inotropes in 12 (34.2%) patients. Twenty-five (71.4%) patients were discharged without any sequelae; six (17%) patients were discharged with neurological sequelae such as vegetative state, seizures, and focal deficits; and four (11.4%) patients died.

## Discussion

Periodic epidemics of acute encephalitis occur in India, resulting in substantial mortality and morbidity, primarily caused by viruses. Although JE is the most prevalent cause, there is a rise in the number of cases of encephalitis caused by other viruses [7].

Out of 35 cases, in 22 (62.8%) cases, viral isolates were identified, while 13 (37.2%) cases had unidentified pathogens. These unidentified cases may be the result of other pathogens or factors, such as autoimmune encephalitis. Meligy et al. [8] and Beig et al. [9] also could not identify viruses in over 50% of patients, similar to our study. In this study, mumps was the most frequently identified virus in eight (22.8%) cases, which is likely attributable to the recent rise in mumps cases in India [10]. Among the other viruses found were dengue in four (11.4%) cases, HSV and EBV in two cases (5.7%) each, and a few others such as H1N1, COVID-19, adenovirus, rabies virus, and rotavirus in a single case (2.8%) each. Unlike other studies, we did not identify any cases of Japanese encephalitis [11].

The male preponderance was substantial (3.4:1), which is in line with the findings of other studies such as those of Kumar et al. [12] and Tripathy et al. [13]. The school-age group was the most significantly impacted, with infants and toddlers/preschoolers following in that order. The majority of respiratory infections were observed in school-age children, which is likely attributable to the presence of close contact at school. This underscores the necessity of infection control measures and vaccinations. Dengue cases were concentrated in the 10-12 age group, whereas mumps cases were dispersed across the 4-12 age group. Kamble and Raghvendra [14] and Chakrabarti et al. (2022) [15] conducted studies that also demonstrated distinct age-group impacts.

The region with the maximum number of cases was Chikhali, followed by Bhosari, Chinchwad, and Nigdi. This may be attributable to the concentration of migration labor in those regions. There was no significant correlation between the socioeconomic and nutritional status of patients in various etiological categories of viral encephalitis.

The most prevalent symptom was fever seen in 32 (91.4%) patients, seizures in 22 (62.9%) patients, vomiting in 19 (54.3%) patients, and altered mental status in 15 (42.9%) patients, following in that order. These results are in alignment with the research conducted by Venkatesan et al. [16]. Mumps encephalitis was characterized by cheek swelling, whereas dengue encephalitis cases showed a maculopapular rash. Dengue, EBV, H1N1, and HSV cases exhibited severe presentations, in line with the literature suggesting higher morbidity and mortality rates for these viruses. The study involved an uncommon case of rabies encephalitis that was initially misdiagnosed as a result of the parents' concealment of a dog bite history. Despite the patient's survival, the fulminant course resulted in severe neurological sequelae. Hemachudha et al. [17] and Despond et al. [18] have observed that rabies in children can manifest in a variety of ways, which can complicate the diagnosis.

Polio, MR, and rotavirus vaccines, which are included in the Universal Immunisation Programme (UIP) and are administered at no cost through the government health system, have been administered to the majority of patients. The MMR vaccine was not administered to any of the patients. The exclusion of mumps from the Universal Immunisation Programme (UIP) schedule may be the reason for the current resurgence of mumps cases. Consequently, it was the most prevalent viral isolate identified in the investigation. The reintroduction of the mumps vaccine into the UIP is the primary focus of this study. Abu Bashar et al. [10] also emphasized this aspect of the study.

It has been noted that patients with severe GCS scores are typically associated with increased mortality rates, with a statistical significance of P=0.017. Patients with dengue, H1N1, EBV, and unknown etiologies exhibited low GCS scores. Kneen et al. [19] also found that children with a Glasgow Coma Scale (GCS) score below 8 had substantially higher rates of intubation and poorer neurological outcomes than those with higher GCS scores, similar to our research. Twelve individuals in our sample reported experiencing shock. Among the patients, seven cases of unknown etiology, one patient with mumps, and patients with dengue, H1N1, EBV, and HSV, severe shock was observed. All of them required ionotropic support, with only a small number requiring mechanical ventilation. The P-value for the difference in shock incidence between survivors and non-survivors was 0.068, indicating that it is a significant prognostic factor. In agreement with our research, Solomon et al. [20] emphasized that the combined impact of diminished blood flow and inflammation on the brain frequently results in worse outcomes in patients with encephalitis. It is imperative to promptly identify and address shock in order to increase the likelihood of survival and minimize neurological impairment. The most prevalent central nervous examination findings in 14 subjects were signs of meningeal irritation, including neck rigidity, Kernig's sign, and Brudzinski's sign. Focal deficits were observed in six patients, cranial nerve palsies in five patients, and papilledema in two patients.

In order to promptly initiate and manage patient care, we implemented a combination of clinical and radiological imaging findings, microbiological testing, and other diagnostic tools. Eight cases of mumps, two cases of HSV, and one case of adenovirus were identified by the CSF PCR test. In a rabies case, CSF antibodies were identified with a titer of 1:128. The nasopharyngeal, throat swab, and stool PCR tests were positive for COVID-19, H1N1, rotavirus, and enterovirus, respectively. Patients who contracted dengue and EBV showed antibodies in their serum. In addition, research has shown that routine viral PCR assays frequently fail to detect viruses in cerebrospinal fluid (CSF) due to low viral loads or the presence of viruses in brain tissue rather than CSF [21].

**Figure 3:**
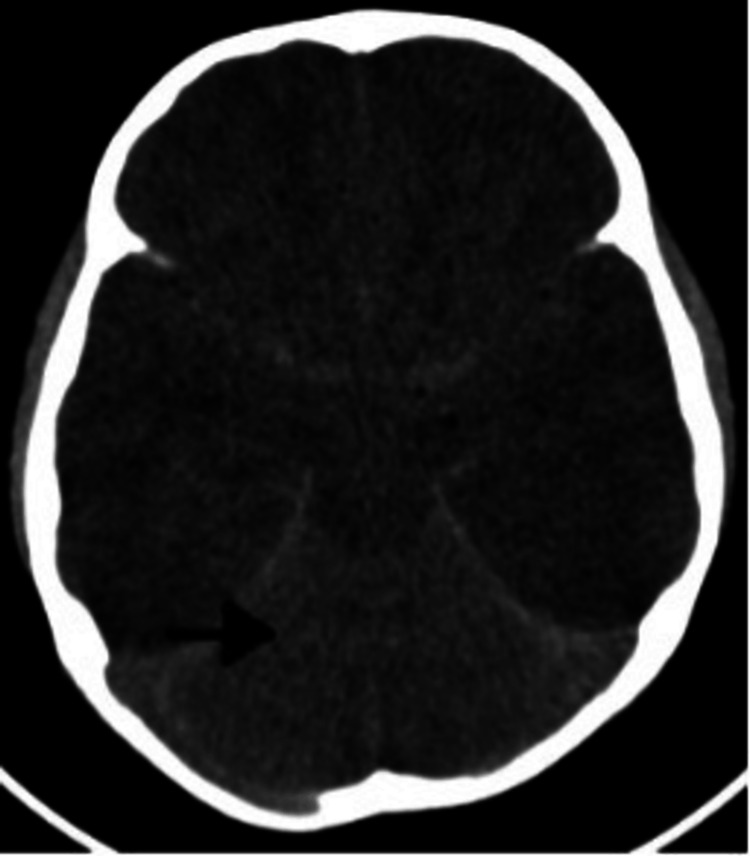
CT image of an H1N1 encephalitis patient in this study showing "white cerebellum sign," which is a poor prognostic indicator CT: computed tomography

Two patients showed wave deceleration on the EEG, while four patients experienced epileptiform discharges. On MRI, 14 patients showed hyperintense subcortical lesions, seven had cortical involvement, five had both, and six had normal findings. A "white cerebellum sign" on a CT scan is a poor prognosticator, as it indicates extensive cerebral edema and severe hypoxic injury, as demonstrated in a single patient with H1N1 encephalitis in our study (Figure 3) [22,23]. This is in accordance with the research conducted by Dahamou et al. [22] and Krishnan and Chowdhury [23]. In contrast to previous investigations (Rona et al. [24] and Haktanir [25]), the MRI of the patient referenced above demonstrated heterogenous irregular enhancements and hemorrhagic alterations, which is an atypical finding.

**Figure 4:**
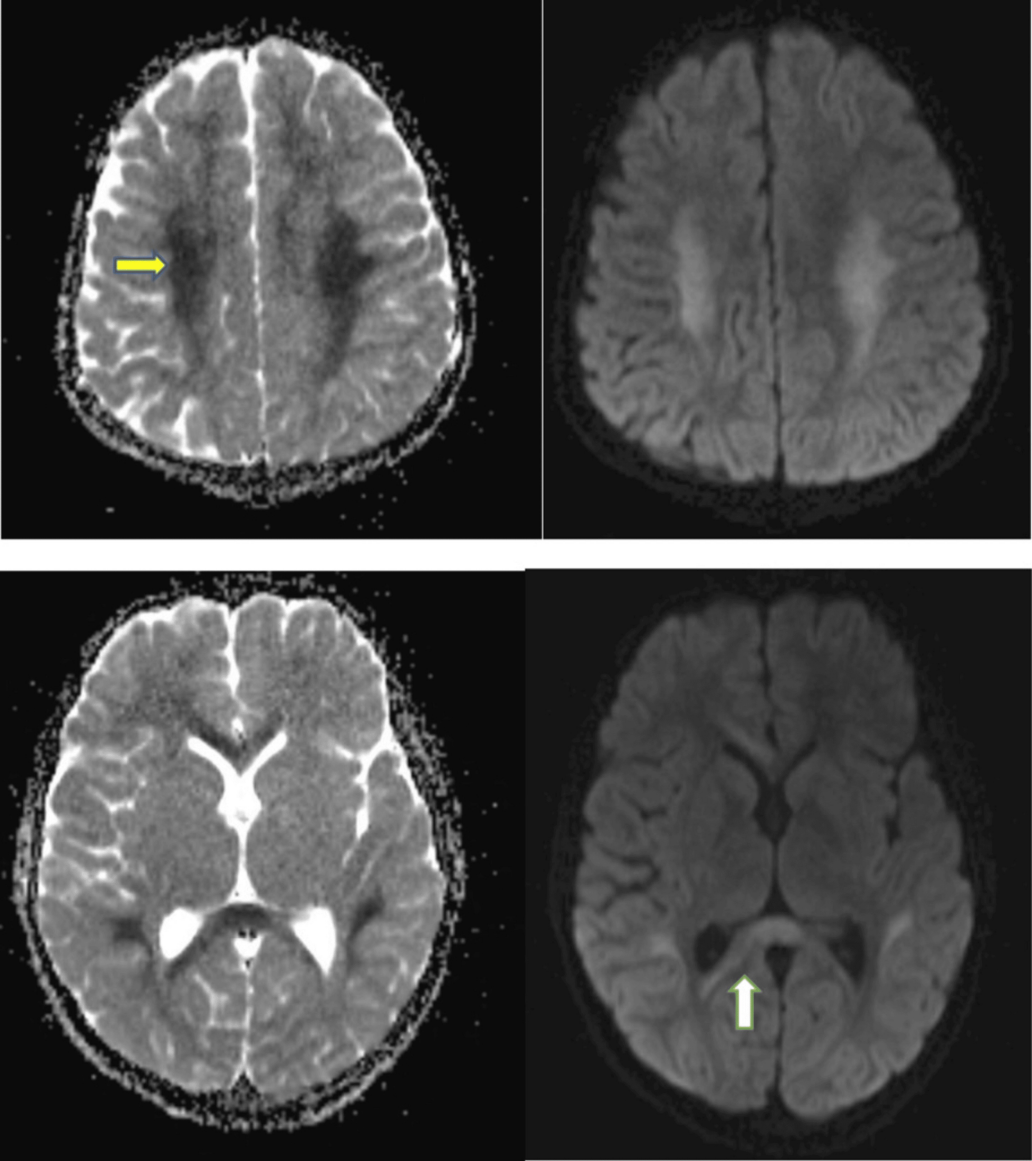
MRI ADC axial section images of a patient in our study showing "involvement of corpus callosum predominantly splenium" in a case of MERS caused by EBV MRI: magnetic resonance imaging, ADC: apparent diffusion coefficient, MERS: mild encephalitis/encephalopathy with reversible splenium lesion, EBV: Epstein-Barr virus

Figure 4 illustrates how our study identified mild encephalitis/encephalopathy with reversible splenium lesion (MERS) based on large altered signal intensity areas in the corpus callosum, periventricular matter, parietal white matter, and corona radiata following EBV infection. Tada et al. [26] described MERS in the context of various other viral illnesses.

**Figure 5:**
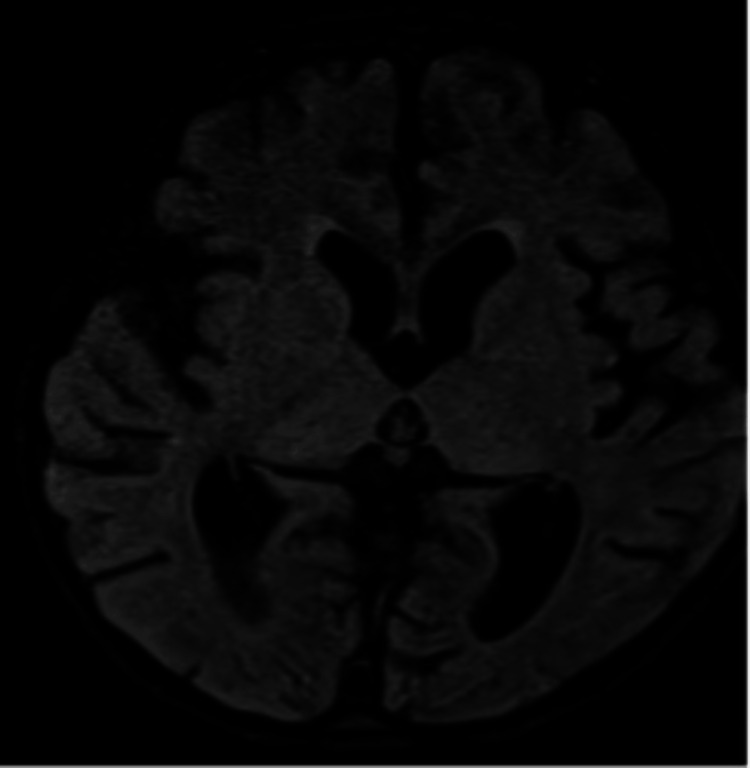
MRI showing involvement of thalami and diffuse cerebral atrophy in a case of rabies encephalitis included in our study All three MRI pictures were of participants included in our study. MRI: magnetic resonance imaging

MRI of the rabies encephalitis patient, as shown in Figure 5, demonstrated hyperintensities in bilateral peri-trigonal and bilateral deep frontal white matter, non-specific white matter alterations, bilateral thalami, cerebellum, and ventral side of the pons, and cerebral atrophy, a classic finding.

Thirty-five (100%) subjects needed PICU admission, antiedema, antibiotics, and acyclovir, followed by steroids and inotropes in 12 (34.2%) patients. The recovery rate without sequelae was in 25 (71.4%) patients; sequelae such as altered mental state (vegetative state) seen in rabies and H1N1 encephalitis, seizures, nerve palsies, and focal deficit were present in four patients, mainly cases of HSV encephalitis and EBV encephalitis. Mortality was seen in one patient with dengue encephalitis with severe shock and in three patients with unknown etiology.

Overall, individuals with dengue, HSV, EBV, and unknown etiologies experienced a fulminant course. Despite being the most commonly isolated virus, mumps had a favorable course and outcome when compared to others.

The clinical course of the rabies encephalitis patient, a three-year-old male child, began with a presentation of fever, cough, respiratory distress, altered sensorium, and multiple seizures. Initial assessments indicated severe neurological impairment, with a Glasgow Coma Scale score of 7/15, signs of respiratory distress, autonomic fluctuations, and positive pyramidal and extrapyramidal findings. Differential diagnoses included tubercular meningitis, viral encephalitis (with potential causes such as parainfluenza, adenoviral, enteroviral, and Epstein-Barr virus), mycoplasma encephalitis, autoimmune encephalitis, and acute demyelination syndrome. Extensive investigations were conducted, including blood tests, MRI, lumbar puncture, and various antibody screenings. Initial treatments involved antibiotics, antivirals, intravenous immunoglobulin (IVIG), methylprednisolone, and supportive care. Despite these interventions, the patient's condition did not improve, and symptoms such as dystonia and opisthotonic posturing worsened. A history of a scratch from a rabid dog led to the consideration of rabies encephalitis, which was confirmed by the presence of rabies antibodies in the CSF. MRI findings showed progressive cerebral and cerebellar atrophy. The patient was shifted to the ward after a 21-day stay in the PICU. The patient received comprehensive care, including physiotherapy, anti-dystonic medications, nutritional support, and frequent repositioning. The patient was started on amantadine based on the evidence from case reports. He remained in a persistent vegetative state with severe neurological deficits and intermittent autonomic disturbances with a modified Rankin score of 5. This is the fifth documented case from Maharashtra and the ninth case from India who survived rabies [27]. It highlights the tragic occurrence of the disease despite vaccination and immunoglobulin administration.

The study limitations were that being a hospital-based study done in a tertiary care center, the incidence and etiological distribution observed in this study may not reflect the actual incidence and etiological distribution of viral encephalitis in the entire population. The CSF viral panel performed by NIV, Pune, does not universally detect all viruses. In most cases, the cause of the disease could not be identified. Follow-up was done only for a shorter period; the long-term assessment was not studied unlike other studies by Gupta et al. [28].

## Conclusions

Despite Japanese encephalitis being historically predominant, our findings highlight a rising prevalence of other viral causes, particularly mumps, which emerged as the most common virus in this cohort. Males were found to be more affected by viral encephalitis than females. Our study also reveals a high incidence among school-age children and also geographic clustering of cases in regions with high labor migration such as Chikhali in the Pimpri Chinchwad Municipal Corporation (PCMC) area. GCS score, need for ventilation, and incidence of shock at the time of admission in correlation to the outcome of the patients were found to be statistically significant. Poor clinical course and outcomes were seen in patients affected with rabies, H1N1 influenza, HSV, and EBV, and very few patients with dengue encephalitis.

## References

[1] Misra UK, Kalita J (2022). Changing spectrum of acute encephalitis syndrome in India and a syndromic approach. Ann Indian Acad Neurol.

[2] Granerod J, Crowcroft NS (2007). The epidemiology of acute encephalitis. Neuropsychol Rehabil.

[3] Narain JP, Dhariwal AC, MacIntyre CR (2017). Acute encephalitis in India: an unfolding tragedy. Indian J Med Res.

[4] (2024). The Times of India: Encephalitis to be made 'notifiable disease': Govt. http://timesofindia.indiatimes.com/city/delhi/encephalitis-to-be-made-notifiable-disease-govt/articleshow/53651028.cms.

[5] Kabilan L (2004). Control of Japanese encephalitis in India: a reality. Indian J Pediatr.

[6] Bansal A, Singhi SC, Singhi PD, Khandelwal N, Ramesh S (2005). Non traumatic coma. Indian J Pediatr.

[7] Kakoti G, Dutta P, Ram Das B, Borah J, Mahanta J (2013). Clinical profile and outcome of Japanese encephalitis in children admitted with acute encephalitis syndrome. Biomed Res Int.

[8] Meligy B, Kadry D, Draz IH, Marzouk H, El Baroudy NR, El Rifay AS (2018). Epidemiological profile of acute viral encephalitis in a sample of Egyptian children. Open Access Maced J Med Sci.

[9] Beig FK, Malik A, Rizvi M, Acharya D, Khare S (2010). Etiology and clinico-epidemiological profile of acute viral encephalitis in children of western Uttar Pradesh, India. Int J Infect Dis.

[10] Abu Bashar MD, Ahmed Khan I, Sridevi G (2024). Recent surge in mumps cases in India: need for urgent remedial measures. Indian Pediatr.

[11] Singh U, Padhi BK, Suresh V, Jindal H, Sah R (2023). Emergence of Japanese encephalitis in nonendemic regions of India: a public health concern?. Ann Med Surg (Lond).

[12] Kumar VS, Sivasubramanian S, Padmanabhan P (2023). Etiological profile and clinico epidemiological patterns of acute encephalitis syndrome in Tamil Nadu, India. J Glob Infect Dis.

[13] Tripathy SK, Mishra P, Dwibedi B, Priyadarshini L, Das RR (2019). Clinico-epidemiological study of viral acute encephalitis syndrome cases and comparison to nonviral cases in children from eastern India. J Glob Infect Dis.

[14] Kamble S, Raghvendra B (2016). A clinico-epidemiological profile of acute encephalitis syndrome in children of Bellary, Karnataka, India. Int J Community Med Public Health.

[15] Chakrabarti SK, Das S, Das P, Debbarma SK (2022). Clinical profile and short term outcome of acute encephalitis syndrome in children: an observational study from a tertiary care centre, Tripura, India. J Clin Diagn Res.

[16] Venkatesan A, Tunkel AR, Bloch KC (2013). Case definitions, diagnostic algorithms, and priorities in encephalitis: consensus statement of the international encephalitis consortium. Clin Infect Dis.

[17] Hemachudha T, Wacharapluesadee S, Mitrabhakdi E, Wilde H, Morimoto K, Lewis RA (2005). Pathophysiology of human paralytic rabies. J Neurovirol.

[18] Despond O, Tucci M, Decaluwe H, Grégoire MC, S Teitelbaum J, Turgeon N (2002). Rabies in a nine-year-old child: the myth of the bite. Can J Infect Dis.

[19] Kneen R, Michael BD, Menson E (2012). Management of suspected viral encephalitis in children - Association of British Neurologists and British Paediatric Allergy, Immunology and Infection Group national guidelines. J Infect.

[20] Solomon T, Hart IJ, Beeching NJ (2007). Viral encephalitis: a clinician's guide. Pract Neurol.

[21] Alam AM, Easton A, Nicholson TR, Irani SR, Davies NW, Solomon T, Michael BD (2023). Encephalitis: diagnosis, management and recent advances in the field of encephalitides. Postgrad Med J.

[22] Dahamou M, Elfarissi MA, Dehneh Y, Aldabbas M, Khoulali M, Oulali N, Moufid F (2022). The white cerebellum sign with good prognosis: a case report. Radiol Case Rep.

[23] Krishnan P, Chowdhury SR (2014). “White cerebellum” sign - a dark prognosticator. J Neurosci Rural Pract.

[24] Rona G, Arifoğlu M, Günbey HP, Yükselmiş U (2021). Influenza A (H1N1)-associated acute necrotizing encephalopathy with unusual posterior reversible encephalopathy syndrome in a child. SN Compr Clin Med.

[25] Haktanir A (2010). MR imaging in novel influenza A(H1N1)-associated meningoencephalitis. AJNR Am J Neuroradiol.

[26] Tada H, Takanashi J, Barkovich AJ (2004). Clinically mild encephalitis/encephalopathy with a reversible splenial lesion. Neurology.

[27] Mani RS, Damodar T, S D (2019). Case reports: Survival from rabies: case series from India. Am J Trop Med Hyg.

[28] Gupta S, Singh AK, Sharma B, Khan IA (2023). Clinical manifestations and disability after acute encephalitis syndrome among pediatric patients in eastern Uttar Pradesh: a retrospective analysis. Cureus.

